# The pupillary light reflex (PLR) as a marker for the ability to work or drive – a feasibility study

**DOI:** 10.1186/s12995-021-00330-2

**Published:** 2021-09-07

**Authors:** Andrea Kaifie, Martin Reugels, Thomas Kraus, Michael Kursawe

**Affiliations:** 1 Institute for Occupational, Social, and Environmental Medicine, Medical Faculty, RWTH Aachen University, Pauwelsstrasse 30, 52074 Aachen, Germany; 2 Department of Medical Statistics, Medical Faculty, RWTH Aachen University, Pauwelsstrasse 30, 52074 Aachen, Germany

**Keywords:** Occupational safety, Responsiveness, Ability to concentrate, Substance abuse

## Abstract

**Background:**

The PLR (pupillary light reflex) can be a marker for pathological medical conditions, such as neurodegenerative or mental health disorders and diseases as well as marker for physiological alterations, such as age, sex or iris color. PLR alterations have been described in people after alcohol consumption, as well. However, the effect of sleep deprivation on PLR parameters is still under debate.

**Methods:**

The aim of this study was to investigate the feasibility of PLR measurements in sleep-deprived and alcohol-exposed participants. In addition, we wanted to identify PLR parameters that were altered by sleep deprivation and alcohol exposure.

**Results:**

Altogether *n* = 50 participants have been included in this study. Differences in the PLR parameters initial diameter (d_init_), latency (∆t_lat_), acceleration (∆t_a_), contraction velocity (ϑ_con_), quarter dilatation velocity (ϑ_1/4dil_), half dilatation time (∆t_1/2_), and the line integral (L(0.3500)) have been evaluated between baseline, sleep deprivation, as well as alcohol exposure. In a generalized linear mixed models design, we could observe statistically significant associations between the type of exposure and the PLR parameters half dilatation time and half dilatation time after the first light pulse (all *p* < 0.05). The participants’ latency showed a significant association in dependence of the type of exposure after the second light pulse (*p* < 0.05).

**Conclusion:**

Our study delivers first promising results to further develop devices that may identify conditions that impair the ability to work or drive.

**Supplementary Information:**

The online version contains supplementary material available at 10.1186/s12995-021-00330-2.

## Background

The ability to work or to drive properly can be influenced by several factors, such as a lack of concentration or responsiveness. In particular, impaired cognitive conditions can elevate the risk of accidents, both at the workplace or on the road. One major driver for accidents is fatigue, in particular, after long working shifts [1, 2]. To avoid accidents that are caused by sleepiness, several tools and methods have been evaluated. Such tools are, for example, specific questionnaires (e.g. Stanford Sleeping Scale), tests that evaluate the concentration and responsiveness (e.g. the psychomotor vigilance task, PVT) or tests that detect physiological markers of fatigue, such as the pupillary unrest index (PUI) [3–5]. The PUI has already been evaluated in drivers during traffic controls. Peters et al. determined the PUI in 137 truck drivers during a police check. The authors came to the conclusion that the PUI is a robust tool with a high technical practicability [6]. Wilhelm had similar findings in his cohort of 1180 truck drivers. He recommended to test the PUI during pauses or traffic controls to avoid sleepiness behind the wheel [7]. However, the determination of the PUI consists of an 11 min recording of the pupil diameter during which the driver is forced to pause. Besides the PUI, Rozanowski et al. determined parameters of the pupillary light reflex (PLR) in order to investigate the level of fatigue [8]. The authors identified parameters of the PLR that were altered by fatigue and concluded, that the measurement of the PLR might be a useful tool to estimate fatigue, but quite faster in comparison to the PUI determination. A further study proved that the area under the curve of the PLR is associated with subjective sleepiness during a 24 h assessment [9].

The PLR is triggered by a light stimulus. If a light stimulus enters the eye, a muscle contraction adapts the pupil size to the light conditions in the surrounding. The constriction parameters of the PLR are dependent on the potential of the sphincter muscle, the function of the retinal photoreceptor cells as well as the afferent and efferent pathways [10]. When stimulated by light, the rods and cones in the retina hyperpolarize resulting in a signal transmission. The intrinsically photosensitive retinal ganglion cells (ipRGCs) have regulatory activity in the PLR by integrating the signals from rods and cones as well as by melanopsin phototransduction. The ipRGCs also contribute to the post-illumination pupil response and are sensitive to light with a wavelength of 482 nm (blue). The constriction is mediated by parasympathetic neurons. Subsequently, sympathetic neurons suppress the parasympathetic innervation of the pupil sphincter which results in a relaxation of the sphincter muscle. In addition, the excitation of the sympathetic pathway leads to a contraction of the sphincter dilatation muscle [10, 11].

There are several factors and conditions that can influence parameters of the PLR. In particular, age has been identified as a strong factor to alter the PLR [12–14]. Fotiou et al. could observe age-dependent effects on pupil size, maximum constriction velocity and acceleration while the latency remained unchanged [12]. Sex and iris color were described as further parameters with the potential to alter the PLR, as well [15, 16]. Besides physiological variations, there are several pathological conditions that have the potential to influence the PLR. Disorders of the pupil, of the parasympathetic pathway as well as of the iris can impair the light response and therefore alter the PLR [17]. There are several further medical preconditions that can lead to an abnormal PLR. In patients with mental health disorders, such as general anxiety disorders [18] or schizophrenia [19] and neurodegenerative diseases, such as Alzheimer [20–24] or Parkinsons disease [9, 25–28], significant differences in PLR parameters have been observed in comparison to healthy subjects.

So far, some studies already investigated the PLR as an indicator for fatigue showing divergent results. While two studies detected PLR alterations in relation to the level of fatigue [8, 29], another study could not find any effects [30]. Fatigue, as well as well as drunkenness, are both drivers for accidents. Studies showed, that persons who consumed alcohol also showed PLR alterations [31, 32]. Both, fatigue as well as alcohol consumption can lead to dangerous situations by a cognitive impairment, in particular where high-level of attention is needed [33, 34]. A low-threshold, valid but fast method in order to identity such a cognitive impairment would be helpful to reduce accidents, both at the workplace and in private life.

The aim of this study was to investigate the feasibility of PLR measurements in sleep-deprived and alcohol-exposed participants. In addition, we wanted to identify parameters that were altered by sleep deprivation and alcohol exposure. The main goal of this analysis was to assess if PLR measurements can be used as a robust and valid marker to determine levels of fatigue and alcohol consumption.

## Materials and methods

### Participants

Altogether *n* = 50 healthy participants were included in this feasibility study (*n* = 25 male, n = 25 female). Exclusion criteria were participants with medical preconditions, such as epilepsy, neurological or psychiatric disorders or diseases, severe eye-diseases (e.g. glaucoma, artificial eye lens), or the use of medication that effects concentration or responsiveness. All participants gave their written informed consent and were willing to participate in all three study visits. The health status of the participants was assessed using questionnaires and a physical examination. The Ethics Committee of the RWTH Aachen University approved this study (EK 054–19).

### Study design

**Fig. 1:**
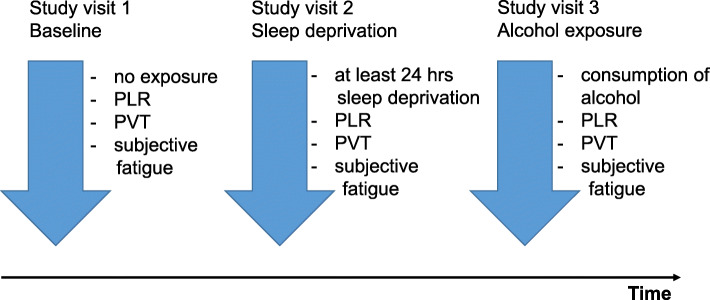
Single-armed study with n = 50 participants, three study visits (baseline, sleep deprivation, alcohol exposure)

This feasibility study was initiated as a single arm exposure study. Measurements of the pupillary light reflex were carried out at three different study visits: (i) a baseline measurement, (ii) after sleep deprivation for at least 24 h, and (iii) after the consumption of a specific amount of alcohol (Fig. 1). The delay in between the different study visits was approximately 7 days for the participants. To avoid a potential circadian confounding, all measurements were carried out in the morning. For each study visit, participants were questioned regarding sleeping disorders, sleeping behavior in the last week, use of medication, and consumption of coffee or tea. In addition, the participants were asked for their subjective level of sleepiness using an ordinal scale (0 = fully awake, 10 = maximal sleepy). Concentration and responsiveness have been objectified using the psychomotor vigilance task (PVT). To assess the patients’ adherence during sleep deprivation, all participants were supervised in the premises of our Institute. For the alcohol exposure, vodka was consumed in dependence on sex, weight, height, and age using the standard operating procedure of the German Aerospace Center [35]. The amount of alcohol consumed was calculated in dependence on gender, age, height, and weight. First, we calculated the total body water (TBW), according to Watson [36]:

TBW_male_ = 2.447–0.09516 x age + 0,1074 x height [cm] + 0.3362 x weight [kg].

TBW_female_ = − 2.097 + 0.1069 x height [cm] + 0.2466 x weight [kg].

Afterwards, the concentration of pure alcohol was calculated with the following formula:

m_alc_ = (target BAC + 0.075‰) x TBW / 0.8 [g] (target BAC: target blood alcohol concentration).

The quantity of the alcoholic beverage to be consumed was calculated, accordingly.

m_beverage_ = m_alc_ × 100 / ε_alcohol-%_ × 0.79 [g] (m_beverage_: quantity of alcoholic beverage in gramm).

(ε_alcohol-%:_ volume fraction of alcohol in the beverage [%])

Participants’ alcohol concentration was determined using a calibrated breath alcohol analyzer 45 min after the consumption (Alcotest, Draeger).

### PLR parameters

In this study, a stationary PLR device (Stellar i-ris, Stellar DBS, Huerth, Germany) was used. Pupillary videos were sampled with 1000 frames per second at a resolution of 1920 × 1080 px. Light pulses were applied to the left eye, the recordings were carried out on the contralateral eye measuring the consensual PLR. Each measurement started with a 3000 ms darkness adaptation period. Thereafter, four light pulses with a duration of 17 ms were applied at 0 ms, 400 ms, 1650 ms, and 2050 ms. Light pulses one and two had a centre wavelength of 625 nm (red light) and were categorized as flashgroup 1, light pulses three and four had a centre wavelength of 468 nm (blue light) and were categorized as flashgroup two. All light pulses were narrow-banded and had an photon flux of 85519E+ 14 [photons/(cm^2^s)] for red light and a photon flux of 273994E+ 15 [photons/(cm^2^s)] of blue light at the retina. Altogether, this series of four light pulses was applied 4 times consecutively per study visit and within a period of 10 min. All parameters were presented as averages of the repeated measurements. The following parameters were calculated from the raw data:

*Initial diameter****d***_***init***_: is defined as the average initial diameter of the pupil at the time of the first light pulse.

*Latency****∆t***_***lat***_: describes the time between the light pulse and the following initial contraction of the pupil.

*Acceleration****∆t***_***a***_: is defined as a point on the curve after the light impulse where acceleration is maximal.

*Contraction velocity****ϑ***_***con***_: describes the mean contraction velocity.

*Quarter dilatation velocity****ϑ***_***1/4dil***_: describes the initial dilatation velocity of the pupil and is a marker for the steepness of the curve.

*Half dilatation time****∆t***_***1/2***_: is described as the dilatation time until the pupil has dilatated by half of the contraction amplitude.

*Line integral****L(0.3500)*:** describes the length of the curve.

**Fig. 2:**
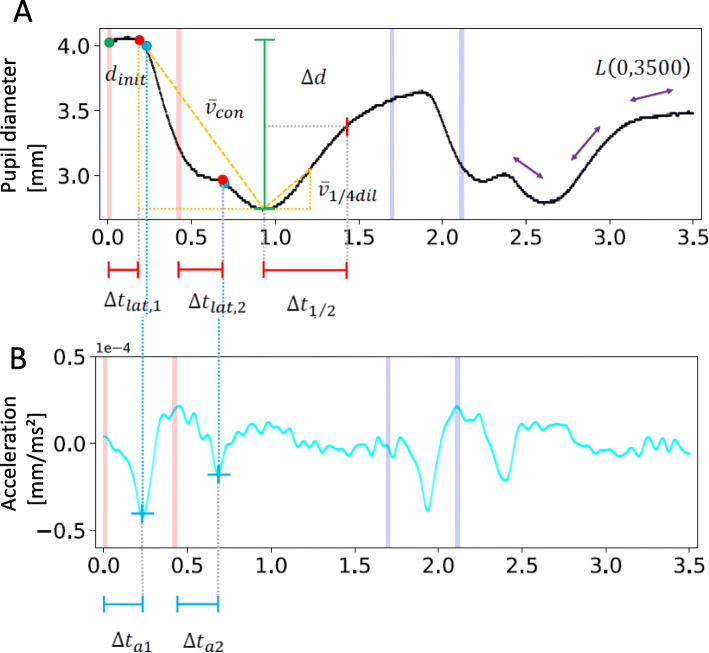
Illustration of the different PLR parameters in the PLR plot. The upper illustration (A) describes the pupil diameter over time and the corresponding parameters that could be estimated form the curve: initial diameter (***d***_***init***_), latency (***∆t***_***lat***_), contraction velocity (***ϑ***_***con***_), quarter dilatation velocity (***ϑ***_***1/4dil***_), half dilatation time (***∆t***_***1/2***_) and line integral (***L(0.3500)***). The lower picture (B) illustrates the acceleration of the pupil with its corresponding parameter (***∆t***_***a***_)

All parameters are illustrated in Fig. 2.

### Statistical analyses

Participants’ characteristics were collected and analysed using SAS Software (SAS 9.4, SAS Institute Inc., Cary, NC, USA.). Descriptive analyses of demographic data, preconditions, and lifestyle habits, such as smoking or sleeping behavior have been carried out. For further testing, the values for acceleration, quarter dilatation velocity, and half dilatation time have been logarithmised to achieve a normal distribution of the corresponding residuals. PVT mean values were calculated by excluding lapses (RTs ≥500 ms) and responses faster than 150 ms. Correlation analyses between potential confounding factors and PLR parameters have been conducted. One-way ANOVAs were used to describe the distribution of PLR parameters as continuous variables between the different study visits (baseline vs. sleep deprivation vs. alcohol exposure) while the assumption of homoscedasticity was fulfilled. To assess differing effects in dependence of the applied wavelength on the participants’ eye a comparison of PLR parameter alterations between red and blue light exposure was carried out. For each above mentioned parameter a separate mixed model with two respectively three repeated factors has been designed, which included potential confounding factors, such as sex, age, eye color, hyperopia/myopia, astigmatism, or smoking status. In addition, analyses comparing PLR parameter alteration after the first and the second light pulse were carried out. Influence diagnostics and sensitivity analyses were performed to achieve a stable, converging model. Important interaction effects were included to control for corresponding effects. Ultimately, a significance level of *p* = 0.05 was used to detect the significant independent variables. Repeated measurements were considered in the model as a covariate as well as a random term.

## Results

Altogether *n* = 50 participants have been included in this study. Demographic data, sleeping behavior and medical preconditions are summarized in Table 1. Participants had a mean age of 42.6 years (range 18–68 years). Fifty percent of the study population were female (*n* = 25). More than 70 % (*n* = 37) of the participants had light eyes (blue, grey, or green) and a refractive error (n = 37). Twelve percent of the population (*n* = 6) reported sleeping disorders, in particular sleep maintenance insomnia. The subjective level of fatigue at the baseline was indicated with a mean value of 2.45 (SD 1.84). After an average of 25.74 h sleep deprivation, a mean subjective level of fatigue with 6.42 (SD 1.25) has been documented. After the consumption of a specific amount of vodka, a mean breath alcohol concentration of 0.43 mg/L (SD 0.1 mg/L) has been reached.

**Table 1:** Demographic data, medical preconditions, and sleeping behavior of the study

Parameter	
Age (mean; SD)	42.6 (14.6)
Female sex (n; %)	25 [37]
Light eye colour (n; %)	37 (50)
Myopia/hyperopia (n; %)	37 (74)
Astigmatism (n, %)	16 (32)
Smoking (n; %)	9 (18)
Sleeping disorders (n; %)	6 (12)
Subjective fatigue* (mean, SD)	
- Baseline	2.45 (1.84)
- Sleep deprivation	6.42 (2)
Hours awake during sleep deprivation (mean, SD)	25.74 (1.25)
Breath alcohol concentration in mg/L (mean, SD)	0.43 (0.1)

Population (* rated as ordinal scale (0–10))

Differences in the PLR parameters initial diameter (d_init_), latency (∆t_lat_), acceleration (∆t_a_), contraction velocity (ϑ_con_), quarter dilatation velocity (ϑ_1/4dil_), half dilatation time (∆t_1/2_), and the line integral (L(0.3500)) have been evaluated between the three types of exposure: baseline, sleep deprivation, as well as alcohol exposure (Table 2). For the PLR parameters latency, contraction velocity, quarter dilatation velocity, as well as half dilatation time, a statistically significant difference (*p* < 0.05) could be observed between the type of exposure. Participants showed a longer latency when they were sleep deprived (∆t_lat_ = 226.92 ms, SD 22.52 ms) in comparison to the baseline (∆tlat = 226.66 ms, SD 22.49 ms). Participants who consumed alcohol showed a shorter latency (∆t_lat_ = 224.7 ms, SD 20.93 ms).

**Table 2:** Mean value and standard deviation for PLR parameters

Endpoint (mean, SD)	Baseline	Sleep deprivation	Alcohol exposure	***p***-value*
Initial diameter (pix)	242.79 (48.11)	244.21 (53.6)	244.96 (51.19)	0.23
Latency (ms)	226.23 (22.49)	226.92 (22.52)	224.7 (20.93)	**< 0.05**
Acceleration (ms)	249.66 (29.39)	250.86 (30.78)	250.27 (29.69)	0.35^#^
Contraction velocity (pix/ms)	0.1061 (0.025)	0.1081 (0.029)	0.1091 (0.026)	**< 0.05**
Quarter dilatation velocity (pix/ms)	0.069 (0.015)	0.068 (0.016)	0.0689 (0.015)	**< 0.05**^**#**^
Half dilatation time (ms)	451.05 (104.02)	482.92 (116.76)	483.09 (115.02)	**< 0.05**^**#**^
Line integral	3524.51 (7.27)	3525.35 (8.1)	3525.09 (7.27)	0.096
PVT (ms)	340.83 (50.1)	370.25 (40.62)	357.6 (46.79)	**< 0.05**

*One-way ANOVA; ^#^the values have been logarithmized for the testing procedure

PVT = Psychomotor Vigilance Test

For the parameter contraction velocity, both types of exposure, sleep deprivation (ϑ_con_ = 0.1081, SD 0.029) as well as alcohol consumption (ϑ_con_ = 0.1091, SD 0.026) lead to a higher contraction velocity in comparison to the baseline (ϑ_con_ = 0.1061, SD 0.025). In contrast, participants who were sleep deprived (ϑ_1/4dil_ = 0.068, SD 0.016) or were exposed to alcohol (ϑ_1/4dil_ = 0.0689, SD 0.015) showed a lower mean quarter dilatation velocity in comparison to the baseline (ϑ_1/4dil_ = 0.069, SD 0.015).

The parameter half dilatation time was significantly longer in participants with sleep deprivation (***∆t***_***1/2***_ = 482.92 ms, SD 116.76 ms) and alcohol consumption (***∆t***_***1/2***_ = 483.09 ms, SD 115.02 ms) in comparison to the baseline (***∆t***_***1/2***_ = 451.05 ms, SD 104.02 ms). In the psychomotor vigilance task (PVT), a statistically significant difference could be observed in the response time at the baseline (340.83 ms), in comparison to sleep deprivation (370.25 ms) and after exposure to alcohol (357.6 ms).

**Fig. 3:**
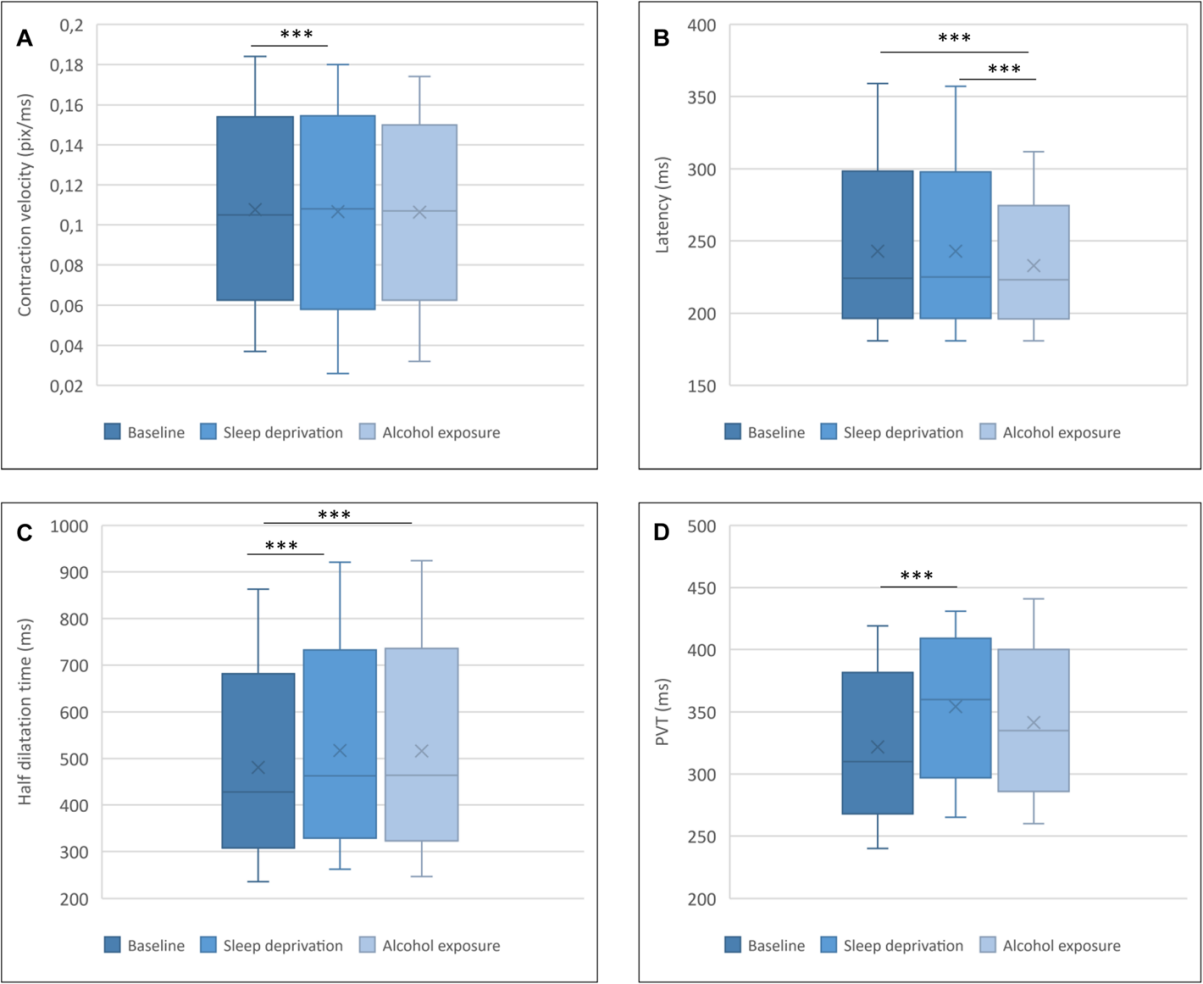
Boxplot of the PLR parameters contraction velocity, latency, half dilation time, and the PVT that were significant in the one-way ANOVA. For each parameter, minimum, first quartile (Q1), median, third quartile (Q3), and maximum were illustrated in dependence of the exposure (baseline vs. sleep deprivation vs. alcohol exposure). Statistically significant differences between the different types of exposure were indicated

In order to assess, from which exposure the differences were caused, a posthoc Tukey-Kramer test was applied and visualized in Fig. 3. A statistically significant difference could be observed between baseline and sleep deprivation for the PLR parameter contraction velocity. For latency, a statistically significant difference could be detected between baseline and sleep deprivation and between sleep deprivation and alcohol exposure. No significant different could be observed between baseline and alcohol exposure. For the PLR parameter half dilatation time, significant differences between baseline and sleep deprivation as well as baseline and alcohol exposure could be found.

In order to assess the effect of the two different colors that were applied, red and blue, we carried out subanalyses for PLR parameter alterations in dependence of the wave length (Table 3).

**Table 3:** Mean value and standard deviation for PLR parameters in dependence of red or blue light exposure during the PLR measurements

	Flashgroup 1: red light				Flashgroup 2: blue light			
Endpoint (mean, SD)	Base-line	Sleep deprivation	Alcohol exposure	p-value*	Base-line	Sleep deprivation	Alcohol exposure	p-value*
Initial diameter (pix)	251.55 (51.17)	253.63 (57.55)	254.19 (54.61)	0.64	233.91 (43.04)	234.69 (47.45)	235.63 (45.64)	0.78
Latency (ms)	223.79 (24.05)	226.38 (25.01)	222.48 (22.19)	**< 0.05**	228.71 (20.5)	227.46 (19.69)	226.95 (19.33)	0.09
Acceleration (ms)	244.13 (29.36)	246.2 (31.25)	244.99 (29.79)	0.33	255.27 (28.36)	255.59 (29.56)	255.64 (28.61)	0.86
Contraction velocity (pix/ms)	0.1061 (0.025)	0.1081 (0.029)	0.1091 (0.026)	**< 0.05**	0.088 (0.022)	0.089 (0.024)	0.087 (0.022)	**< 0.05**
Quarter dilatation velocity (pix/ms)	0.069 (0.015)	0.068 (0.016)	0.069 (0.015)	0.13	0.064 (0.014)	0.064 (0.015)	0.063 (0.014)	**< 0.05**
Half dilatation time (ms)	451.06 (104.02)	482.92 (116.76)	483.09 (115.02)	**< 0.05**	360.5 (59.52)	374.21 (61.07)	367.33 (54.23)	**< 0.05**
Line integral	3524.51 (7.26)	3535.35 (8.05)	3525.08 (7.27)	0.05	3524.51 (7.26)	3535.35 (8.05)	3525.08 (7.27)	0.05

*One-way ANOVA

In summary, red light application led to significant differences in latency, contraction velocity, as well as half dilatation time. For blue light application, additional differences could be observed for quarter dilatation velocity, while latency was near the significance limit.

To gain an overview about the influence of age, personal characteristics, sleeping conditions, and alcohol exposure on the PLR parameters, we added a correlation matrix (Table 4). Overall, a moderate inverse correlation for age and contraction velocity (− 0.23), age and initial diameter (− 0.24), age and line integral (− 0.3), as well as age and quarter dilatation velocity (− 0.37) could be detected. For age and acceleration (0.35) as well as age and latency (0.33), a positive correlation was observed. For smokers, an inverse correlation could be detected for the parameters line integral (− 0.2) and quarter dilatation velocity (− 0.21). A positive correlation could be observed for the breath alcohol concentration and the initial diameter (0.19) and the line integral (0.2). An inverse correlation could be detected or sleeping disorder and the parameters line integral (− 0.21) and quarter dilatation velocity (− 0.2). (Inverse) correlation for all other parameters remained weak.

**Table 4:** Correlation analyses between age, personal characteristics, sleeping conditions, alcohol exposure and PLR parameters

	Acceler-ation	Contraction velocity	Half dilatation time	Initial diameter	Latency	Line integral	Quarter dilatation velocity
**Age**	0.35	−0.23	0.12	−0.24	0.33	−0.30	−0.37
**Light eye color**	0.08	−0.04	0.04	− 0.15	0.10	− 0.05	− 0.11
**Myopia/hyperopia**	0.08	0.02	0.06	0.04	0.11	0.03	−0.02
**Astigmatism**	−0.12	0.16	0.01	0.10	−0.08	0.21	0.16
**Smoking**	0.19	−0.11	0.11	0.07	0.17	−0.20	−0.21
**Breath alcohol**	0.00	0.13	0.12	0.19	−0.01	0.20	0.11
**Sleeping disorder**	−0.09	−0.19	− 0.04	−0.12	− 0.07	−0.21	− 0.20
**Hours awake**	0.04	−0.05	0.02	−0.12	0.04	−0.09	−0.06
**Subjective fatigue**	−0.02	−0.03	− 0.02	−0.05	0.02	−0.01	− 0.03

Afterwards, we used the method of generalised linear mixed models in order to identify the independent effect of sleep deprivation and the consumption of alcohol on PLR parameter alterations. We added potential influencing variables, such as type of exposure (baseline vs. sleep deprivation vs. alcohol exposure), repeated measurements, sex, age, light eyes, refractive error, astigmatism, and smoking into a model to test for fixed effects. When the PLR parameter showed a (narrowly) significant association to the type of exposure, we also tested for levels of significance for PLR parameter alterations in dependence of the light pulse (after first light pulse and after second light pulse). Although, the correlation analyses revealed a moderate association between age and the different PLR parameters, in the test for fixed effect, age narrowly missed the significance threshold in all models. Except for light eyes in the latency, all other parameters, such as sex, refractory error, astigmatism or smoking did not significantly affect PLR parameters. We could observe statistically significant associations between the type of exposure and the PLR parameter half dilatation time after the first light pulse (all *p* < 0.05)(Table 5). The participants’ latency showed a significant association in dependence of the type of exposure after the second light pulse (p < 0.05).

**Table 5:** Generalized linear mixed models for PLR parameters, estimating the effect of exposure (sleep deprivation, alcohol exposure) vs. baseline. Parameters and type of exposure that showed significant effects in the fixed effects model were included. An additional analysis on effects after first or second light pulse was carried out (only significant results are shown)

Endpoint	Parameter	B	S (B)	p-value
Latency	Sleep deprivation	0.98	0.92	0.29
	Alcohol exposure	1.62	0.91	0.08
Latency after second light pulse	Sleep deprivation	3.6	0.72	**< 0.05**
	Alcohol exposure	1.75	0.71	**< 0.05**
	Light eyes	−0.89	3.0	0.77
Half dilatation time	Sleep deprivation	0.050	0.013	**< 0.05**
	Alcohol exposure	−0.044	0.013	**< 0.05**
Half dilatation time after first light pulse	Sleep deprivation	0.064	0.01	**< 0.05**
	Alcohol exposure	−0.068	0.01	**< 0.05**
Contraction velocity	Sleep deprivation	0.001	0.002	0.71
	Alcohol exposure	−0.001	0.002	0.8
Quarter dilatation velocity	Sleep deprivation	−0.02	0.01	0.29
	Alcohol exposure	0.02	0.01	0.36

## Discussion

In this study, we were able to identify PLR parameters that were affected by both, sleep deprivation as well as alcohol exposure. Here, the latency, defined as the time between the light pulse and the following initial contraction of the pupil and the half dilatation time, described as the dilatation time after the contraction until the pupil dilatated by half of the contraction amplitude, showed a statistically significant association between type of exposure and PLR parameter alterations. The PVT confirmed the cognitive impairment of the participants, both being sleep deprived or exposed to alcohol with a significant prolongation of the response time in the test. Besides statistical analyses, another important aim of our study was to determine the participants’ acceptance of the PLR measurements. All participants tolerated well the repetitive application of light pulses to the eye, no adverse effects have been reported during the study.

Although, age, sex or the color of the iris have been described as influencing factors on PLR parameters [12–16], we mainly could determine age-dependent effects. However, the type of exposure as well as the repeated measurements were stronger factors for PLR alterations in our analyses. Sleep deprivation significantly prolonged the latency as well as the dilatation time, while alcohol exposure showed divergent results. The latency was shortened on average while the half dilatation time was prolonged, in accordance with the sleep deprivation in our participants. Our findings are in contrast to the study of Ranzijn et al., who could not observe any effects of sleepiness on PLR parameters [30]. One reason for the different findings could lay in the high-resolution recording of the PLR in our study with 1000 frames per second. With such a technique, we might be able to capture also slight differences in the pupillary response. Those differences may not be detectable in PLR devices that show a lower recording precision. In particular, the PLR parameter alterations latency and half dilatation time that remained significant in the mixed model and that confirm independent effects caused by the type of exposure in contrast to other studies, could strengthen the high-resolution method.

As described in the methods section, we used sixteen repetitions. A hypothesis we pursued was to create a similar level of excitation in the eye after the first light stimulus. After reaching this similar level of excitation, we expected to observe more PLR parameter alterations in dependence of the type of exposure. The identification of PLR alterations after the following light pulses was a main goal of this specific study design. We could observe that the latency of the pupillary contraction showed significant differences in particular between the types of exposure after the second light stimulus in our mixed model analyses. Against our expectation, this parameter was the only one that confirmed our hypothesis. The half dilatation time was on average significant and after the first light stimulus.

In addition, we expected to observe more homogenous results in terms of constriction and dilatation. The dominance of one pathway, the sympathetic or the parasympathetic, would not lead to a prolongation of constriction accompanied with an extended dilatation period and vice versa. In contrast to that, we observed both, a prolongation of constriction together with an extended dilatation period in sleep-deprived participants which is physiologically contrary. This was in contrast to participants with alcohol exposure, where the latency was reduced while the half dilatation time was prolonged. This speaks in favor of a dominance of the parasympathetic pathway.

For alcohol, PLR parameter alterations have been described in relation to the alcohol concentration before. While lower alcohol breath concentration led to an increased peak constriction period, higher alcohol concentrations had a contrary effect with a decreased constriction amplitude and velocity [10, 32, 34, 38]. Hall et al. explained the impaired pupillary response with an inhibition of the parasympathetic and hence, dominance of the sympathetic system [10, 39]. Interestingly, also long-term PLR effects by chronic alcohol abuse have been described. Rubin observed different pupillary responses between chronic alcoholics and non-alcoholics [40]. Kvamme et al. even used the PLR as a predictor for relapse in detoxified alcohol-dependent patients [41].

The PLR has been widely analysed for several physiological and pathological conditions. For instance, the pupillary response in healthy subjects can be affected by the intensity of the light stimulus [42]. Seasonal as well as circadian effects or intraindividual characteristics, such as ipRGC polymorphism may play a role in PLR parameter variations [29, 43, 44]. Bitsios et al. described in their study, that an altered initial pupil diameter and a decrease of the PLR amplitude was associated with the anticipation of an (adverse) stimulus [45]. As indicated in the introduction, several health conditions are associated with PLR alterations. In particular, in patients with traumatic brain injuries [46, 47], affective disorders or autism [48–50] PLR parameter changes have been described. In particular, centrally acting medications can modify the PLR, depending on the sympathetic or parasympathetic activity [11, 37]. All of these studies underline the influenceability of PLR parameters.

The main goal of this analyses was to identify PLR parameters alterations with a high-resolution device caused by sleep deprivation or the exposure to alcohol. Overall, we could observe PLR parameter alterations caused by sleep deprivation as well as alcohol exposure. We identified two parameters, that were significantly affected by the type of exposure – the latency and the half dilatation time. In sleep-deprived patients, a prolongation of the latency as a constriction parameter and the half dilatation time as a dilatation parameter could be observed. For participants that consumed alcohol, a reduction of the latency and a prolongation of the half dilatation time was detected. In the mixed model, both type of exposure led to independent effects on both PLR parameters. The identification of such parameters could help to improve low-threshold testing of people in potentially at-risk situations, such as truck drivers, pilots, or surgeons. In particular, fatigue is a main driver for (fatal) accidents and human errors. Our analyses might be a first step in the development of PLR devices that support the estimation of the level of fatigue und hence, the decision to take a regenerative break.

### Limitations

Our study has several limitations. The sleep deprivation has not been carried out under full-experimental and standardized conditions with a defined exposure to light, for example. In the statistical analyses, the repeated measurements showed a significant contribution to PLR parameter alterations. Applied repetitive light pulses to the eye could have led to an independent effect on PLR parameters. We also expected to observe more PLR parameter alterations after the second light pulse. The first light pulse was initially intended to create a similar level of excitation in the eye in order to observe the main alterations after the second light pulse. However, only for the constriction latency a statistically significant difference between sleep deprivation, exposure to alcohol and the baseline has been observed. In addition, a stationary PLR device is in its current form not practical for real-life test, such as next to traffic roads or at a (mobile) workplace. If further studies proof the validity of the measurement procedure, a mobile PLR device with a quick and safe evaluation unit must be developed.

## Supplementary Information

**Additional file 1.**

## Data Availability

The datasets used and/or analysed during the current study are available from the corresponding author on reasonable request.
