# Supplementary material for: The pupillary light reflex (PLR) as a marker for the ability to work or drive – a feasibility study
Source: J Occup Med Toxicol. 2021 Sep 7;16:39. doi: 10.1186/s12995-021-00330-2 (PMC8422642; doi:10.1186/s12995-021-00330-2)
Supplement: Supplementary file 1 — Additional file 1. [file 12995_2021_330_MOESM1_ESM.docx]

**Supplemental Table 1**

| **subject** | **exposure** | **variable** | **mean** | **SD** |
| --- | --- | --- | --- | --- |
| **1** | baseline | Initial diameter (pix)  Latency (ms)  Acceleration (ms)  Contraction velocity (pix/ms)  Quarter dilatation velocity (pix/ms)  Half dilatation time (ms)  Line integral | 191.48  231.5  253.07  0.067  0.0504  395.41  3516.45 | 24.36  16.98  15.63  0.019  0.0099  99.71  3.44 |
| **1** | sleep deprivation | Initial diameter (pix)  Latency (ms)  Acceleration (ms)  Contraction velocity (pix/ms)  Quarter dilatation velocity (pix/ms)  Half dilatation time (ms)  Line integral | 226.89  232.765  261.81  0.092  0.064  454.72  3523.20 | 26.85  15.55  18.71  0.019  0.009  107.93  3.861 |
| **1** | alcohol exposure | Initial diameter (pix)  Latency (ms)  Acceleration (ms)  Contraction velocity (pix/ms)  Quarter dilatation velocity (pix/ms)  Half dilatation time (ms)  Line integral | 213.45  239.83  271.96  0.074  0.049  461.04  3517.37 | 27.67  18.4  20.03  0.024  0.009  164.34  3.29 |
| **2** | baseline | Initial diameter (pix)  Latency (ms)  Acceleration (ms)  Contraction velocity (pix/ms)  Quarter dilatation velocity (pix/ms)  Half dilatation time (ms)  Line integral | 222.27  217.08  234.72  0.086  0.062  386.07  3520.69 | 24.86  13.61  17.64  0.013  0.006  72.8  2.38 |
| **2** | sleep deprivation | Initial diameter (pix)  Latency (ms)  Acceleration (ms)  Contraction velocity (pix/ms)  Quarter dilatation velocity (pix/ms)  Half dilatation time (ms)  Line integral | 230.14  224.48  237.12  0.092  0.0637  397.66  3523.92 | 29.28  12.72  18.62  0.015  0.006  77.46  3.87 |
| **2** | alcohol exposure | Initial diameter (pix)  Latency (ms)  Acceleration (ms)  Contraction velocity (pix/ms)  Quarter dilatation velocity (pix/ms)  Half dilatation time (ms)  Line integral | 225.35  218.97  235.67  0.094  0.0669  401.17  3523.57 | 28.34  12.92  17.73  0.015  0.006  89.43  3.21 |
| **3** | baseline | Initial diameter (pix)  Latency (ms)  Acceleration (ms)  Contraction velocity (pix/ms)  Quarter dilatation velocity (pix/ms)  Half dilatation time (ms)  Line integral | 215.43  217.03  229.3  0.106  0.076  360.94  3528.04 | 28.55  14.64  16.98  0.016  0.007  53.29  2.97 |
| **3** | sleep deprivation | Initial diameter (pix)  Latency (ms)  Acceleration (ms)  Contraction velocity (pix/ms)  Quarter dilatation velocity (pix/ms)  Half dilatation time (ms)  Line integral | 239.95  213.0625000  234.5507813  0.125  0.082  414.72  3534.37 | 40.95  12.62  23.13  0.017  0.006  73.91  4.18 |
| **3** | alcohol exposure | Initial diameter (pix)  Latency (ms)  Acceleration (ms)  Contraction velocity (pix/ms)  Quarter dilatation velocity (pix/ms)  Half dilatation time (ms)  Line integral | 217.19  208.95  223.7  0.116  0.079  378.22  3532.95 | 33.64  14.4  18.66  0.019  0.006  72.18  4.52 |
| **4** | baseline | Initial diameter (pix)  Latency (ms)  Acceleration (ms)  Contraction velocity (pix/ms)  Quarter dilatation velocity (pix/ms)  Half dilatation time (ms)  Line integral | 211.9929032  222.5645161  236.2496774  0.0667885  0.0449486  388.7407407  3513.35 | 21.9588009  17.7990799  12.4580041  0.0139244  0.0101851  98.2862944  2.2557140 |
| **4** | sleep deprivation | Initial diameter (pix)  Latency (ms)  Acceleration (ms)  Contraction velocity (pix/ms)  Quarter dilatation velocity (pix/ms)  Half dilatation time (ms)  Line integral | 213.5275000  222.8888889  239.0725000  0.0710789  0.0482577  413.9000000  3513.93 | 23.0910890  16.5081711  13.0829061  0.0140773  0.0105844  114.3815648  1.5921242 |
| **4** | alcohol exposure | Initial diameter (pix)  Latency (ms)  Acceleration (ms)  Contraction velocity (pix/ms)  Quarter dilatation velocity (pix/ms)  Half dilatation time (ms)  Line integral | 215.8664063  216.8412698  236.2815625  0.0761825  0.0533243  379.2812500  3517.19 | 24.4810885  15.3414959  15.7423745  0.0144615  0.0098675  93.7505079  4.4711880 |
| **5** | baseline | Initial diameter (pix)  Latency (ms)  Acceleration (ms)  Contraction velocity (pix/ms)  Quarter dilatation velocity (pix/ms)  Half dilatation time (ms)  Line integral | 321.9262500  224.8125000  251.0943750  0.1001889  0.0707869  403.5666667  3523.77 | 35.9202966  12.6727742  19.2986844  0.0219562  0.0090804  87.3235493  1.2578037 |
| **5** | sleep deprivation | Initial diameter (pix)  Latency (ms)  Acceleration (ms)  Contraction velocity (pix/ms)  Quarter dilatation velocity (pix/ms)  Half dilatation time (ms)  Line integral | 329.3212500  222.2968750  248.2681250  0.1124697  0.0758433  465.7931034  3528.07 | 42.8141682  13.8457256  15.0010956  0.0238999  0.0084004  123.3202589  1.8228731 |
| **5** | alcohol exposure | Initial diameter (pix)  Latency (ms)  Acceleration (ms)  Contraction velocity (pix/ms)  Quarter dilatation velocity (pix/ms)  Half dilatation time (ms)  Line integral | 343.4465625  221.1093750  250.7264063  0.1042113  0.0690350  482.8437500  3524.96 | 42.0034426  12.5320126  19.1799429  0.0241513  0.0084745  125.7463078  2.3541976 |
| **6** | baseline | Initial diameter (pix)  Latency (ms)  Acceleration (ms)  Contraction velocity (pix/ms)  Quarter dilatation velocity (pix/ms)  Half dilatation time (ms)  Line integral | 325.7627419  224.2542373  256.7565574  0.1173687  0.0828817  413.2333333  3532.50 | 38.1280494  13.8869738  24.0489550  0.0179850  0.0082438  85.9261344  2.4793406 |
| **6** | sleep deprivation | Initial diameter (pix)  Latency (ms)  Acceleration (ms)  Contraction velocity (pix/ms)  Quarter dilatation velocity (pix/ms)  Half dilatation time (ms)  Line integral | 330.2678125  219.2222222  250.4964063  0.1230003  0.0847797  448.3125000  3533.43 | 45.8186751  16.0336787  21.5685951  0.0193149  0.0081541  119.4182825  5.3502249 |
| **6** | alcohol exposure | Initial diameter (pix)  Latency (ms)  Acceleration (ms)  Contraction velocity (pix/ms)  Quarter dilatation velocity (pix/ms)  Half dilatation time (ms)  Line integral | 298.5723438  221.7777778  250.2439062  0.1031700  0.0764061  402.0000000  3526.37 | 34.0877856  14.3745344  18.1380935  0.0186036  0.0063555  140.6052491  3.2592650 |
| **7** | baseline | Initial diameter (pix)  Latency (ms)  Acceleration (ms)  Contraction velocity (pix/ms)  Quarter dilatation velocity (pix/ms)  Half dilatation time (ms)  Line integral | 234.1662500  222.5555556  243.1254688  0.1182304  0.0811802  392.2812500  3533.41 | 29.6872605  21.0458622  21.7050796  0.0212425  0.0091724  62.8610129  6.6181719 |
| **7** | sleep deprivation | Initial diameter (pix)  Latency (ms)  Acceleration (ms)  Contraction velocity (pix/ms)  Quarter dilatation velocity (pix/ms)  Half dilatation time (ms)  Line integral | 248.6470313  225.5156250  248.5931746  0.1299654  0.0807245  478.2187500  3535.95 | 34.6015297  16.0267556  21.5896441  0.0174158  0.0061554  81.7727142  3.2302314 |
| **7** | alcohol exposure | Initial diameter (pix)  Latency (ms)  Acceleration (ms)  Contraction velocity (pix/ms)  Quarter dilatation velocity (pix/ms)  Half dilatation time (ms)  Line integral | 232.8731250  220.2500000  243.6690476  0.1252298  0.0829258  415.4687500  3536.01 | 33.3830148  17.5309251  23.7708739  0.0160206  0.0073456  50.5248544  4.2961287 |
| **8** | baseline | Initial diameter (pix)  Latency (ms)  Acceleration (ms)  Contraction velocity (pix/ms)  Quarter dilatation velocity (pix/ms)  Half dilatation time (ms)  Line integral | 308.3637500  207.4375000  221.8662500  0.0895817  0.0702148  354.1785714  3523.28 | 34.5241018  13.4434441  14.6509645  0.0219141  0.0134148  152.1712083  4.5590736 |
| **8** | sleep deprivation | Initial diameter (pix)  Latency (ms)  Acceleration (ms)  Contraction velocity (pix/ms)  Quarter dilatation velocity (pix/ms)  Half dilatation time (ms)  Line integral | 313.9592187  208.6349206  225.8918750  0.0968095  0.0713904  435.5806452  3525.18 | 33.8543831  11.5971376  17.4801689  0.0170678  0.0113338  155.5356212  3.1607681 |
| **8** | alcohol exposure | Initial diameter (pix)  Latency (ms)  Acceleration (ms)  Contraction velocity (pix/ms)  Quarter dilatation velocity (pix/ms)  Half dilatation time (ms)  Line integral | 337.3176563  208.5937500  227.1520312  0.0942873  0.0710212  412.8333333  3524.01 | 28.1762553  13.0514473  17.7501091  0.0158482  0.0086445  135.5076040  2.7097904 |
| **9** | baseline | Initial diameter (pix)  Latency (ms)  Acceleration (ms)  Contraction velocity (pix/ms)  Quarter dilatation velocity (pix/ms)  Half dilatation time (ms)  Line integral | 278.0287500  227.9375000  255.4251563  0.0925103  0.0689239  378.0312500  3523.01 | 25.5899821  14.2904952  22.6286162  0.0141442  0.0055570  59.4319589  1.6397610 |
| **9** | sleep deprivation | Initial diameter (pix)  Latency (ms)  Acceleration (ms)  Contraction velocity (pix/ms)  Quarter dilatation velocity (pix/ms)  Half dilatation time (ms)  Line integral | 261.9420313  225.7343750  260.3315625  0.0891419  0.0634268  417.6774194  3519.84 | 26.7452813  12.6519240  23.2594653  0.0171139  0.0058968  101.7800222  2.0420852 |
| **9** | alcohol exposure | Initial diameter (pix)  Latency (ms)  Acceleration (ms)  Contraction velocity (pix/ms)  Quarter dilatation velocity (pix/ms)  Half dilatation time (ms)  Line integral | 281.6932813  226.2343750  252.5831250  0.0980425  0.0698672  399.8125000  3525.98 | 24.8360234  14.9472535  25.3090172  0.0185550  0.0075954  66.7458756  3.0610577 |
| **10** | baseline | Initial diameter (pix)  Latency (ms)  Acceleration (ms)  Contraction velocity (pix/ms)  Quarter dilatation velocity (pix/ms)  Half dilatation time (ms)  Line integral | 224.1687097  234.0327869  259.9430645  0.0985489  0.0657311  385.9354839  3525.13 | 24.1011349  16.5690145  27.8963054  0.0142574  0.0075381  57.2111049  2.7632398 |
| **10** | sleep deprivation | Initial diameter (pix)  Latency (ms)  Acceleration (ms)  Contraction velocity (pix/ms)  Quarter dilatation velocity (pix/ms)  Half dilatation time (ms)  Line integral | 226.5775000  231.1093750  259.8367188  0.1073661  0.0691156  419.4838710  3526.79 | 27.3686261  19.0688953  31.0791121  0.0129230  0.0049837  54.7787628  1.8982665 |
| **10** | alcohol exposure | Initial diameter (pix)  Latency (ms)  Acceleration (ms)  Contraction velocity (pix/ms)  Quarter dilatation velocity (pix/ms)  Half dilatation time (ms)  Line integral | 229.6325000  224.7187500  258.3425000  0.1117135  0.0690739  442.4375000  3529.71 | 28.3088240  16.2641574  29.0066764  0.0130608  0.0045254  67.5465448  2.3843801 |
| **11** | baseline | Initial diameter (pix)  Latency (ms)  Acceleration (ms)  Contraction velocity (pix/ms)  Quarter dilatation velocity (pix/ms)  Half dilatation time (ms)  Line integral | 291.3678125  224.6093750  254.0326563  0.1043608  0.0811040  346.1379310  3527.64 | 36.2719590  15.1454506  25.5106645  0.0116600  0.0074423  39.5454206  2.6111085 |
| **11** | sleep deprivation | Initial diameter (pix)  Latency (ms)  Acceleration (ms)  Contraction velocity (pix/ms)  Quarter dilatation velocity (pix/ms)  Half dilatation time (ms)  Line integral | 276.1080645  222.3548387  248.5870968  0.1074815  0.0779768  399.7857143  3527.83 | 49.3216456  11.5447764  20.2452566  0.0124154  0.0091293  108.4620946  3.2579544 |
| **11** | alcohol exposure | Initial diameter (pix)  Latency (ms)  Acceleration (ms)  Contraction velocity (pix/ms)  Quarter dilatation velocity (pix/ms)  Half dilatation time (ms)  Line integral | 260.3639062  220.7187500  254.5209375  0.1048121  0.0771323  370.2500000  3528.35 | 34.9902272  10.8532326  25.0568166  0.0098275  0.0054584  35.4141035  2.7422591 |
| **12** | baseline | Initial diameter (pix)  Latency (ms)  Acceleration (ms)  Contraction velocity (pix/ms)  Quarter dilatation velocity (pix/ms)  Half dilatation time (ms)  Line integral | 250.6257813  217.2343750  238.6785938  0.0853226  0.0620473  369.8437500  3520.19 | 30.4934202  14.5533961  22.3508847  0.0125772  0.0052846  54.9505459  2.3454231 |
| **12** | sleep deprivation | Initial diameter (pix)  Latency (ms)  Acceleration (ms)  Contraction velocity (pix/ms)  Quarter dilatation velocity (pix/ms)  Half dilatation time (ms)  Line integral | 272.8553125  215.5156250  243.7004688  0.0949741  0.0674533  417.4333333  3522.91 | 30.1381725  13.2808397  22.7621284  0.0124138  0.0047535  77.5749756  1.9502668 |
| **12** | alcohol exposure | Initial diameter (pix)  Latency (ms)  Acceleration (ms)  Contraction velocity (pix/ms)  Quarter dilatation velocity (pix/ms)  Half dilatation time (ms)  Line integral | 237.3901563  215.8437500  245.6973438  0.0803418  0.0558465  385.3437500  3517.78 | 21.8346992  13.3359744  27.0583325  0.0113688  0.0070677  39.6953553  1.9584260 |
| **13** | baseline | Initial diameter (pix)  Latency (ms)  Acceleration (ms)  Contraction velocity (pix/ms)  Quarter dilatation velocity (pix/ms)  Half dilatation time (ms)  Line integral | 188.9826563  234.5312500  253.1743750  0.0753385  0.0529081  372.7812500  3517.95 | 19.3137127  18.2721653  20.3903456  0.0120062  0.0047653  54.1653754  1.3654313 |
| **13** | sleep deprivation | Initial diameter (pix)  Latency (ms)  Acceleration (ms)  Contraction velocity (pix/ms)  Quarter dilatation velocity (pix/ms)  Half dilatation time (ms)  Line integral | 193.6696875  230.0468750  255.0160938  0.0801931  0.0545815  396.5517241  3519.52 | 19.6106887  17.1227406  24.1865471  0.0132717  0.0054395  62.7568889  1.4476191 |
| **13** | alcohol exposure | Initial diameter (pix)  Latency (ms)  Acceleration (ms)  Contraction velocity (pix/ms)  Quarter dilatation velocity (pix/ms)  Half dilatation time (ms)  Line integral | 183.8859375  229.8437500  260.0485938  0.0711643  0.0469568  421.2258065  3516.81 | 16.9991373  20.3120253  22.3284941  0.0124604  0.0044608  65.1574183  1.6887385 |
| **14** | baseline | Initial diameter (pix)  Latency (ms)  Acceleration (ms)  Contraction velocity (pix/ms)  Quarter dilatation velocity (pix/ms)  Half dilatation time (ms)  Line integral | 176.5056250  228.0156250  255.8373438  0.0807407  0.0570843  381.0000000  3519.28 | 16.7455223  19.8841823  30.4454131  0.0083991  0.0033256  40.3893747  1.5294650 |
| **14** | sleep deprivation | Initial diameter (pix)  Latency (ms)  Acceleration (ms)  Contraction velocity (pix/ms)  Quarter dilatation velocity (pix/ms)  Half dilatation time (ms)  Line integral | 181.9189063  226.8593750  247.2462500  0.0831446  0.0593810  381.4062500  3519.61 | 19.5614741  20.3585848  23.4346847  0.0114462  0.0041429  58.5972855  1.7075842 |
| **14** | alcohol exposure | Initial diameter (pix)  Latency (ms)  Acceleration (ms)  Contraction velocity (pix/ms)  Quarter dilatation velocity (pix/ms)  Half dilatation time (ms)  Line integral | 178.7540625  232.5781250  253.0109375  0.0813907  0.0556013  400.5000000  3519.94 | 18.8207067  20.5154256  26.6186673  0.0097196  0.0031237  54.0810679  1.6748090 |
| **15** | baseline | Initial diameter (pix)  Latency (ms)  Acceleration (ms)  Contraction velocity (pix/ms)  Quarter dilatation velocity (pix/ms)  Half dilatation time (ms)  Line integral | 213.0431250  224.6875000  248.4709375  0.0965743  0.0659574  380.8437500  3523.64 | 23.3610683  15.0109748  22.0515041  0.0139562  0.0060259  45.2057795  3.3144774 |
| **15** | sleep deprivation | Initial diameter (pix)  Latency (ms)  Acceleration (ms)  Contraction velocity (pix/ms)  Quarter dilatation velocity (pix/ms)  Half dilatation time (ms)  Line integral | 204.1292188  232.5156250  252.9495313  0.0844054  0.0577254  371.5937500  3519.55 | 28.7795735  17.7924473  29.1104536  0.0179975  0.0075250  69.2952548  4.0520308 |
| **15** | alcohol exposure | Initial diameter (pix)  Latency (ms)  Acceleration (ms)  Contraction velocity (pix/ms)  Quarter dilatation velocity (pix/ms)  Half dilatation time (ms)  Line integral | 209.8317188  220.4218750  244.6412500  0.0926184  0.0618955  397.5937500  3521.94 | 28.2423002  16.1085649  23.6024698  0.0141193  0.0047769  66.8511065  2.8311879 |
| **16** | baseline | Initial diameter (pix)  Latency (ms)  Acceleration (ms)  Contraction velocity (pix/ms)  Quarter dilatation velocity (pix/ms)  Half dilatation time (ms)  Line integral | 171.3793750  227.9062500  249.8746875  0.0814104  0.0536004  405.0000000  3518.76 | 29.4760316  17.1748420  26.5898587  0.0191832  0.0084367  77.2808377  3.2563828 |
| **16** | sleep deprivation | Initial diameter (pix)  Latency (ms)  Acceleration (ms)  Contraction velocity (pix/ms)  Quarter dilatation velocity (pix/ms)  Half dilatation time (ms)  Line integral | 152.5595313  241.4603175  257.6135938  0.0718707  0.0445094  411.4838710  3518.37 | 29.8185382  25.1946137  35.4900224  0.0212537  0.0083940  105.4656718  3.6854959 |
| **16** | alcohol exposure | Initial diameter (pix)  Latency (ms)  Acceleration (ms)  Contraction velocity (pix/ms)  Quarter dilatation velocity (pix/ms)  Half dilatation time (ms)  Line integral | 171.5317187  226.1406250  252.2957813  0.0829761  0.0510074  434.5806452  3519.15 | 21.8644455  18.1299254  26.1873852  0.0178819  0.0069082  86.6130548  1.3207611 |
| **17** | baseline | Initial diameter (pix)  Latency (ms)  Acceleration (ms)  Contraction velocity (pix/ms)  Quarter dilatation velocity (pix/ms)  Half dilatation time (ms)  Line integral | 263.9059375  237.5937500  275.5190625  0.1314869  0.0769467  533.9032258  3536.26 | 30.6891284  20.3014729  30.2469998  0.0222408  0.0055662  92.7889743  3.5878423 |
| **17** | sleep deprivation | Initial diameter (pix)  Latency (ms)  Acceleration (ms)  Contraction velocity (pix/ms)  Quarter dilatation velocity (pix/ms)  Half dilatation time (ms)  Line integral | 233.6495313  243.0476190  277.4825000  0.1147759  0.0722508  482.4193548  3530.08 | 25.4830087  30.3056625  31.8798165  0.0150703  0.0038444  64.4358999  1.4798889 |
| **17** | alcohol exposure | Initial diameter (pix)  Latency (ms)  Acceleration (ms)  Contraction velocity (pix/ms)  Quarter dilatation velocity (pix/ms)  Half dilatation time (ms)  Line integral | 251.6332813  230.0937500  274.3573438  0.1264620  0.0756238  527.0322581  3535.45 | 29.3730932  19.2293746  32.7550218  0.0206888  0.0052033  94.1639602  2.4272837 |
| **18** | baseline | Initial diameter (pix)  Latency (ms)  Acceleration (ms)  Contraction velocity (pix/ms)  Quarter dilatation velocity (pix/ms)  Half dilatation time (ms)  Line integral | 219.5264516  237.0322581  261.1038710  0.0622641  0.0474873  413.1333333  3513.77 | 16.1521820  21.0151918  13.6242526  0.0104112  0.0045830  171.1838606  1.7248328 |
| **18** | sleep deprivation | Initial diameter (pix)  Latency (ms)  Acceleration (ms)  Contraction velocity (pix/ms)  Quarter dilatation velocity (pix/ms)  Half dilatation time (ms)  Line integral | 219.7320313  238.0000000  260.7415625  0.0632611  0.0485617  384.7419355  3513.56 | 18.6227063  18.7574324  13.4911954  0.0071779  0.0080578  104.1190894  1.3415117 |
| **18** | alcohol exposure | Initial diameter (pix)  Latency (ms)  Acceleration (ms)  Contraction velocity (pix/ms)  Quarter dilatation velocity (pix/ms)  Half dilatation time (ms)  Line integral | 230.5698361  232.2833333  254.8428333  0.0759692  0.0593057  375.6333333  3516.84 | 22.4082346  18.0602491  16.9796362  0.0145251  0.0076033  141.1683652  2.5185068 |
| **19** | baseline | Initial diameter (pix)  Latency (ms)  Acceleration (ms)  Contraction velocity (pix/ms)  Quarter dilatation velocity (pix/ms)  Half dilatation time (ms)  Line integral | 262.1792188  223.0156250  248.4675000  0.0996458  0.0744837  358.3548387  3525.69 | 29.3459066  16.5207827  21.1926002  0.0134456  0.0069520  47.9340725  2.5686762 |
| **19** | sleep deprivation | Initial diameter (pix)  Latency (ms)  Acceleration (ms)  Contraction velocity (pix/ms)  Quarter dilatation velocity (pix/ms)  Half dilatation time (ms)  Line integral | 236.4675806  230.2741935  250.0211290  0.0772621  0.0585501  335.6333333  3519.86 | 29.2490319  18.1036808  19.4016399  0.0171946  0.0112966  50.9905165  3.5813052 |
| **19** | alcohol exposure | Initial diameter (pix)  Latency (ms)  Acceleration (ms)  Contraction velocity (pix/ms)  Quarter dilatation velocity (pix/ms)  Half dilatation time (ms)  Line integral | 253.2415625  223.1875000  242.8978125  0.0962457  0.0728795  333.1333333  3524.22 | 23.8429136  12.6162845  18.8954985  0.0130475  0.0066556  34.0306276  3.1994175 |
| **20** | baseline | Initial diameter (pix)  Latency (ms)  Acceleration (ms)  Contraction velocity (pix/ms)  Quarter dilatation velocity (pix/ms)  Half dilatation time (ms)  Line integral | 240.3600000  220.6774194  250.8493548  0.1189547  0.0789831  398.5483871  3531.44 | 33.2769070  13.0820540  24.9337682  0.0123253  0.0065948  43.9208220  2.0673587 |
| **20** | sleep deprivation | Initial diameter (pix)  Latency (ms)  Acceleration (ms)  Contraction velocity (pix/ms)  Quarter dilatation velocity (pix/ms)  Half dilatation time (ms)  Line integral | 246.6965625  223.5781250  256.4420312  0.1261582  0.0807064  437.0000000  3534.15 | 41.5912997  16.9385488  27.1610189  0.0188320  0.0095973  71.6317745  4.9878235 |
| **20** | alcohol exposure | Initial diameter (pix)  Latency (ms)  Acceleration (ms)  Contraction velocity (pix/ms)  Quarter dilatation velocity (pix/ms)  Half dilatation time (ms)  Line integral | 224.3773438  223.7580645  251.0646032  0.1054073  0.0703332  390.1935484  3527.44 | 34.1678402  16.8364218  24.6427844  0.0144045  0.0080247  57.8695080  3.1380887 |
| **21** | baseline | Initial diameter (pix)  Latency (ms)  Acceleration (ms)  Contraction velocity (pix/ms)  Quarter dilatation velocity (pix/ms)  Half dilatation time (ms)  Line integral | 198.2003125  220.4062500  241.6684127  0.0700508  0.0501587  452.3548387  3513.56 | 21.8030213  13.5631606  16.2496459  0.0112027  0.0039474  161.7605087  1.1094223 |
| **21** | sleep deprivation | Initial diameter (pix)  Latency (ms)  Acceleration (ms)  Contraction velocity (pix/ms)  Quarter dilatation velocity (pix/ms)  Half dilatation time (ms)  Line integral | 224.4079688  215.5312500  232.5095313  0.0878316  0.0604646  492.8214286  3519.35 | 31.6739495  15.1111950  17.4495641  0.0105939  0.0052557  178.8010684  1.9112135 |
| **21** | alcohol exposure | Initial diameter (pix)  Latency (ms)  Acceleration (ms)  Contraction velocity (pix/ms)  Quarter dilatation velocity (pix/ms)  Half dilatation time (ms)  Line integral | 220.8921311  234.1967213  249.2003333  0.0718720  0.0505643  445.8148148  3519.67 | 22.4506885  16.5557842  19.3424530  0.0107991  0.0049810  134.8382783  5.2658772 |
| **22** | baseline | Initial diameter (pix)  Latency (ms)  Acceleration (ms)  Contraction velocity (pix/ms)  Quarter dilatation velocity (pix/ms)  Half dilatation time (ms)  Line integral | 260.8514063  230.5468750  260.9014516  0.1157418  0.0764246  412.3000000  3530.43 | 31.3686550  17.8057684  27.4443615  0.0186364  0.0068625  60.9012259  4.2359365 |
| **22** | sleep deprivation | Initial diameter (pix)  Latency (ms)  Acceleration (ms)  Contraction velocity (pix/ms)  Quarter dilatation velocity (pix/ms)  Half dilatation time (ms)  Line integral | 271.9300000  240.3968254  273.1559375  0.1133637  0.0726530  445.3225806  3530.17 | 33.5643248  22.2779652  30.8305242  0.0146473  0.0038910  68.8768857  3.5741899 |
| **22** | alcohol exposure | Initial diameter (pix)  Latency (ms)  Acceleration (ms)  Contraction velocity (pix/ms)  Quarter dilatation velocity (pix/ms)  Half dilatation time (ms)  Line integral | 279.9270313  227.6562500  261.3762500  0.1201763  0.0788520  466.0000000  3530.98 | 35.3824669  19.6995035  26.2320914  0.0230328  0.0064620  102.4255053  2.3304367 |
| **23** | baseline | Initial diameter (pix)  Latency (ms)  Acceleration (ms)  Contraction velocity (pix/ms)  Quarter dilatation velocity (pix/ms)  Half dilatation time (ms)  Line integral | 200.2000000  214.6406250  222.0260937  0.0784767  0.0562554  349.5483871  3519.79 | 22.1986448  13.2513757  16.9404521  0.0120266  0.0064759  45.4109387  3.5049876 |
| **23** | sleep deprivation | Initial diameter (pix)  Latency (ms)  Acceleration (ms)  Contraction velocity (pix/ms)  Quarter dilatation velocity (pix/ms)  Half dilatation time (ms)  Line integral | 185.3868750  213.1702128  212.9642553  0.0677417  0.0502141  339.1304348  3518.34 | 17.5585650  14.7726527  14.6685749  0.0165237  0.0093387  39.4453820  3.0698007 |
| **23** | alcohol exposure | Initial diameter (pix)  Latency (ms)  Acceleration (ms)  Contraction velocity (pix/ms)  Quarter dilatation velocity (pix/ms)  Half dilatation time (ms)  Line integral | 205.2639063  215.2968750  226.1784127  0.0857018  0.0577971  392.2068966  3521.90 | 25.0510909  13.5970114  18.9264045  0.0143984  0.0062091  102.6164309  2.8116584 |
| **24** | baseline | Initial diameter (pix)  Latency (ms)  Acceleration (ms)  Contraction velocity (pix/ms)  Quarter dilatation velocity (pix/ms)  Half dilatation time (ms)  Line integral | 228.9356250  215.0781250  234.4595313  0.1091638  0.0754259  392.1562500  3528.33 | 38.1502627  12.6958369  20.8124631  0.0166744  0.0073456  61.3447760  3.4539264 |
| **24** | sleep deprivation | Initial diameter (pix)  Latency (ms)  Acceleration (ms)  Contraction velocity (pix/ms)  Quarter dilatation velocity (pix/ms)  Half dilatation time (ms)  Line integral | 215.9557813  214.1406250  229.4018750  0.1070681  0.0708143  432.2666667  3527.21 | 36.5396141  12.7127822  18.5357861  0.0229603  0.0093100  110.8074933  3.1826988 |
| **24** | alcohol exposure | Initial diameter (pix)  Latency (ms)  Acceleration (ms)  Contraction velocity (pix/ms)  Quarter dilatation velocity (pix/ms)  Half dilatation time (ms)  Line integral | 212.5107813  213.0468750  233.2734375  0.1030494  0.0667942  423.8387097  3525.84 | 34.2242248  14.6779499  20.5493297  0.0225624  0.0087187  97.9960176  4.6695113 |
| **25** | baseline | Initial diameter (pix)  Latency (ms)  Acceleration (ms)  Contraction velocity (pix/ms)  Quarter dilatation velocity (pix/ms)  Half dilatation time (ms)  Line integral | 276.2391935  226.0000000  248.6522581  0.1320540  0.0896980  394.0689655  3540.69 | 39.2673367  17.9854221  23.2199242  0.0191341  0.0091628  53.1993477  5.1556044 |
| **25** | sleep deprivation | Initial diameter (pix)  Latency (ms)  Acceleration (ms)  Contraction velocity (pix/ms)  Quarter dilatation velocity (pix/ms)  Half dilatation time (ms)  Line integral | 264.3351613  220.8387097  248.2812903  0.1356155  0.0878507  427.3214286  3542.89 | 32.5257540  17.8253766  30.9183592  0.0161748  0.0083714  54.6087263  7.2655506 |
| **25** | alcohol exposure | Initial diameter (pix)  Latency (ms)  Acceleration (ms)  Contraction velocity (pix/ms)  Quarter dilatation velocity (pix/ms)  Half dilatation time (ms)  Line integral | 268.9385714  225.0925926  257.0445455  0.1238122  0.0783681  430.9629630  3534.93 | 33.2955456  15.2108061  25.1993259  0.0170297  0.0089277  68.9930166  3.8782539 |
| **26** | baseline | Initial diameter (pix)  Latency (ms)  Acceleration (ms)  Contraction velocity (pix/ms)  Quarter dilatation velocity (pix/ms)  Half dilatation time (ms)  Line integral | 293.9215625  213.2187500  235.4595313  0.1208762  0.0880999  370.7741935  3533.10 | 32.9786259  14.8638998  18.7668228  0.0183204  0.0078562  54.2101011  4.4057555 |
| **26** | sleep deprivation | Initial diameter (pix)  Latency (ms)  Acceleration (ms)  Contraction velocity (pix/ms)  Quarter dilatation velocity (pix/ms)  Half dilatation time (ms)  Line integral | 309.6192188  209.8281250  236.8048437  0.1326724  0.0899177  434.2500000  3539.35 | 38.6358425  11.5436435  19.0322368  0.0256184  0.0095760  110.7684558  5.0426010 |
| **26** | alcohol exposure | Initial diameter (pix)  Latency (ms)  Acceleration (ms)  Contraction velocity (pix/ms)  Quarter dilatation velocity (pix/ms)  Half dilatation time (ms)  Line integral | 325.9475000  207.8906250  237.6175000  0.1308231  0.0892150  430.0312500  3535.95 | 39.8153875  13.1500705  20.1623883  0.0212038  0.0072091  91.9369986  4.6096762 |
| **27** | baseline | Initial diameter (pix)  Latency (ms)  Acceleration (ms)  Contraction velocity (pix/ms)  Quarter dilatation velocity (pix/ms)  Half dilatation time (ms)  Line integral | 291.1706452  210.7540984  229.0268852  0.1265577  0.0903294  411.4666667  3533.35 | 44.9712456  13.8692174  17.4739453  0.0153549  0.0073621  130.3288496  3.8087298 |
| **27** | sleep deprivation | Initial diameter (pix)  Latency (ms)  Acceleration (ms)  Contraction velocity (pix/ms)  Quarter dilatation velocity (pix/ms)  Half dilatation time (ms)  Line integral | 340.6159375  206.0793651  228.6400000  0.1480767  0.0999258  477.2187500  3542.58 | 54.2826556  12.3488299  18.9870944  0.0143144  0.0066483  113.4869364  3.1855189 |
| **27** | alcohol exposure | Initial diameter (pix)  Latency (ms)  Acceleration (ms)  Contraction velocity (pix/ms)  Quarter dilatation velocity (pix/ms)  Half dilatation time (ms)  Line integral | 321.5746875  207.8593750  231.5342188  0.1427691  0.0946830  448.2812500  3540.33 | 46.1715967  11.8654724  18.2605984  0.0124514  0.0056497  82.9267163  3.2677907 |
| **28** | baseline | Initial diameter (pix)  Latency (ms)  Acceleration (ms)  Contraction velocity (pix/ms)  Quarter dilatation velocity (pix/ms)  Half dilatation time (ms)  Line integral | 285.9725000  220.1406250  245.0392188  0.1226211  0.0823958  416.5312500  3533.75 | 35.0578536  15.7650077  20.6895582  0.0151174  0.0075200  83.9043236  2.9125958 |
| **28** | sleep deprivation | Initial diameter (pix)  Latency (ms)  Acceleration (ms)  Contraction velocity (pix/ms)  Quarter dilatation velocity (pix/ms)  Half dilatation time (ms)  Line integral | 286.7096875  217.5781250  237.3029687  0.1266503  0.0857818  426.3125000  3535.89 | 39.7764789  14.8802678  21.0630332  0.0153046  0.0066402  91.2493314  3.6420940 |
| **28** | alcohol exposure | Initial diameter (pix)  Latency (ms)  Acceleration (ms)  Contraction velocity (pix/ms)  Quarter dilatation velocity (pix/ms)  Half dilatation time (ms)  Line integral | 289.6381250  213.8750000  239.6364063  0.1296011  0.0857723  442.3437500  3536.48 | 39.9517885  14.1056107  21.0654381  0.0221782  0.0098539  106.8571541  6.3481480 |
| **29** | baseline | Initial diameter (pix)  Latency (ms)  Acceleration (ms)  Contraction velocity (pix/ms)  Quarter dilatation velocity (pix/ms)  Half dilatation time (ms)  Line integral | 258.8368750  228.4687500  257.9288889  0.0997003  0.0611702  517.7857143  3523.10 | 27.5500840  24.4299899  36.5994551  0.0148407  0.0053606  91.2396872  2.5102732 |
| **29** | sleep deprivation | Initial diameter (pix)  Latency (ms)  Acceleration (ms)  Contraction velocity (pix/ms)  Quarter dilatation velocity (pix/ms)  Half dilatation time (ms)  Line integral | 258.6161905  228.0000000  257.4610000  0.0988501  0.0622413  495.9285714  3524.03 | 29.0608362  17.8244124  34.5927678  0.0152480  0.0044052  117.6775034  1.7090407 |
| **29** | alcohol exposure | Initial diameter (pix)  Latency (ms)  Acceleration (ms)  Contraction velocity (pix/ms)  Quarter dilatation velocity (pix/ms)  Half dilatation time (ms)  Line integral | 266.7877049  229.9508197  249.4091228  0.0979941  0.0610353  520.7500000  3523.24 | 24.9738941  22.0842525  27.4122570  0.0166354  0.0062146  100.5572770  1.9017019 |
| **30** | baseline | Initial diameter (pix)  Latency (ms)  Acceleration (ms)  Contraction velocity (pix/ms)  Quarter dilatation velocity (pix/ms)  Half dilatation time (ms)  Line integral | 256.4187500  255.2222222  285.2592063  0.0947012  0.0607571  430.6562500  3524.24 | 25.1763857  25.5951384  34.3738715  0.0177493  0.0067540  78.2850345  5.0565497 |
| **30** | sleep deprivation | Initial diameter (pix)  Latency (ms)  Acceleration (ms)  Contraction velocity (pix/ms)  Quarter dilatation velocity (pix/ms)  Half dilatation time (ms)  Line integral | 282.0865625  236.1406250  279.1550000  0.1204297  0.0714193  523.0937500  3529.97 | 33.7363098  17.9380479  34.4749757  0.0186963  0.0071151  127.0173990  3.1506885 |
| **30** | alcohol exposure | Initial diameter (pix)  Latency (ms)  Acceleration (ms)  Contraction velocity (pix/ms)  Quarter dilatation velocity (pix/ms)  Half dilatation time (ms)  Line integral | 259.4926563  237.8906250  272.5264063  0.0967956  0.0628579  443.5161290  3523.43 | 33.9056676  16.9285306  30.2558401  0.0207416  0.0097964  117.1256495  4.4178193 |
| **31** | baseline | Initial diameter (pix)  Latency (ms)  Acceleration (ms)  Contraction velocity (pix/ms)  Quarter dilatation velocity (pix/ms)  Half dilatation time (ms)  Line integral | 260.1896667  205.5000000  223.6265000  0.1206162  0.0873666  396.5336667  3529.90 | 36.4812671  11.8829033  18.3108639  0.0131449  0.0073044  81.1118023  2.6387778 |
| **31** | sleep deprivation | Initial diameter (pix)  Latency (ms)  Acceleration (ms)  Contraction velocity (pix/ms)  Quarter dilatation velocity (pix/ms)  Half dilatation time (ms)  Line integral | 243.6081250  210.1093750  229.3667188  0.1150513  0.0778107  419.6451613  3529.51 | 31.8732764  12.4939569  18.0114900  0.0136462  0.0045844  68.4242064  2.4342307 |
| **31** | alcohol exposure | Initial diameter (pix)  Latency (ms)  Acceleration (ms)  Contraction velocity (pix/ms)  Quarter dilatation velocity (pix/ms)  Half dilatation time (ms)  Line integral | 223.9948438  213.0937500  229.7201563  0.0986796  0.0682206  389.2187500  3523.42 | 27.0754785  12.8936046  18.7334212  0.0095179  0.0044269  43.0213933  1.8060468 |
| **32** | baseline | Initial diameter (pix)  Latency (ms)  Acceleration (ms)  Contraction velocity (pix/ms)  Quarter dilatation velocity (pix/ms)  Half dilatation time (ms)  Line integral | 251.0274194  232.6129032  262.5483871  0.0907553  0.0652767  398.5333333  3522.48 | 20.7729955  17.5558638  23.3879288  0.0150475  0.0057140  55.1919506  2.2710285 |
| **32** | sleep deprivation | Initial diameter (pix)  Latency (ms)  Acceleration (ms)  Contraction velocity (pix/ms)  Quarter dilatation velocity (pix/ms)  Half dilatation time (ms)  Line integral | 229.5375000  238.1562500  264.2641270  0.0763605  0.0573753  361.1333333  3519.24 | 23.1247635  19.6954743  23.0720174  0.0164886  0.0078370  59.6621751  2.0917576 |
| **32** | alcohol exposure | Initial diameter (pix)  Latency (ms)  Acceleration (ms)  Contraction velocity (pix/ms)  Quarter dilatation velocity (pix/ms)  Half dilatation time (ms)  Line integral | 236.9624194  234.6451613  271.1281667  0.0806642  0.0564461  404.3333333  3519.52 | 25.7961183  20.7159531  25.0826611  0.0203871  0.0067667  85.9685923  2.5016227 |
| **33** | baseline | Initial diameter (pix)  Latency (ms)  Acceleration (ms)  Contraction velocity (pix/ms)  Quarter dilatation velocity (pix/ms)  Half dilatation time (ms)  Line integral | 270.6840625  265.3968254  276.4336508  0.0879402  0.0448498  425.0000000  3519.46 | 35.2065907  24.9893782  21.0550187  0.0277476  0.0037859  35.7030766  2.5765560 |
| **33** | sleep deprivation | Initial diameter (pix)  Latency (ms)  Acceleration (ms)  Contraction velocity (pix/ms)  Quarter dilatation velocity (pix/ms)  Half dilatation time (ms)  Line integral | 277.8406250  258.9687500  282.4564063  0.0913376  0.0473250  514.3125000  3519.40 | 36.3542784  27.1211092  24.6792624  0.0234763  0.0037925  90.9233770  1.7546225 |
| **33** | alcohol exposure | Initial diameter (pix)  Latency (ms)  Acceleration (ms)  Contraction velocity (pix/ms)  Quarter dilatation velocity (pix/ms)  Half dilatation time (ms)  Line integral | 264.3779688  250.3281250  271.4761290  0.0831143  0.0426779  520.8421053  3517.81 | 34.2606514  18.6760645  22.4445168  0.0246328  0.0038586  117.6574336  1.6338527 |
| **34** | baseline | Initial diameter (pix)  Latency (ms)  Acceleration (ms)  Contraction velocity (pix/ms)  Quarter dilatation velocity (pix/ms)  Half dilatation time (ms)  Line integral | 211.0823438  210.6562500  230.2806250  0.1249217  0.0777899  439.2666667  3535.87 | 33.7109937  15.5330868  25.7868596  0.0178035  0.0062305  58.2495702  4.7283371 |
| **34** | sleep deprivation | Initial diameter (pix)  Latency (ms)  Acceleration (ms)  Contraction velocity (pix/ms)  Quarter dilatation velocity (pix/ms)  Half dilatation time (ms)  Line integral | 175.2879687  219.5312500  233.2531746  0.0967845  0.0628659  392.7692308  3527.28 | 26.2157004  21.3452115  27.8040197  0.0196936  0.0072901  72.8109600  4.3158238 |
| **34** | alcohol exposure | Initial diameter (pix)  Latency (ms)  Acceleration (ms)  Contraction velocity (pix/ms)  Quarter dilatation velocity (pix/ms)  Half dilatation time (ms)  Line integral | 197.2132813  210.1093750  223.9995082  0.1211322  0.0761940  423.3103448  3533.71 | 32.6458736  18.4357462  25.4580709  0.0204250  0.0065490  76.5222607  3.3609138 |
| **35** | baseline | Initial diameter (pix)  Latency (ms)  Acceleration (ms)  Contraction velocity (pix/ms)  Quarter dilatation velocity (pix/ms)  Half dilatation time (ms)  Line integral | 213.4881250  243.6718750  269.1087500  0.0705712  0.0506627  403.7096774  3516.41 | 27.1205145  18.7151197  21.7432875  0.0136137  0.0082464  139.6410371  3.5530062 |
| **35** | sleep deprivation | Initial diameter (pix)  Latency (ms)  Acceleration (ms)  Contraction velocity (pix/ms)  Quarter dilatation velocity (pix/ms)  Half dilatation time (ms)  Line integral | 165.0206250  252.1406250  259.6818750  0.0424514  0.0330846  346.8750000  3512.02 | 16.9803852  15.2084638  19.0183211  0.0079642  0.0053027  61.6640926  1.5900561 |
| **35** | alcohol exposure | Initial diameter (pix)  Latency (ms)  Acceleration (ms)  Contraction velocity (pix/ms)  Quarter dilatation velocity (pix/ms)  Half dilatation time (ms)  Line integral | 184.3301563  250.1562500  262.1112500  0.0491854  0.0398482  315.3125000  3515.31 | 22.0097009  16.4125569  21.5213123  0.0124070  0.0086626  84.2119162  2.3032785 |
| **36** | baseline | Initial diameter (pix)  Latency (ms)  Acceleration (ms)  Contraction velocity (pix/ms)  Quarter dilatation velocity (pix/ms)  Half dilatation time (ms)  Line integral | 265.3637500  223.5156250  244.2187500  0.1211293  0.0848500  405.4516129  3532.36 | 31.5044227  15.6347113  26.4422109  0.0162724  0.0056648  69.1009424  2.5650099 |
| **36** | sleep deprivation | Initial diameter (pix)  Latency (ms)  Acceleration (ms)  Contraction velocity (pix/ms)  Quarter dilatation velocity (pix/ms)  Half dilatation time (ms)  Line integral | 288.8462500  225.0937500  244.9575000  0.1273843  0.0860397  454.7241379  3533.66 | 41.3937624  18.8492084  28.8358683  0.0171292  0.0074107  83.6051272  2.6951253 |
| **36** | alcohol exposure | Initial diameter (pix)  Latency (ms)  Acceleration (ms)  Contraction velocity (pix/ms)  Quarter dilatation velocity (pix/ms)  Half dilatation time (ms)  Line integral | 280.6529688  214.6875000  239.7629688  0.1332834  0.0889032  448.1562500  3536.60 | 36.0581818  13.9953223  25.6915231  0.0190630  0.0064527  73.5974074  2.0579606 |
| **37** | baseline | Initial diameter (pix)  Latency (ms)  Acceleration (ms)  Contraction velocity (pix/ms)  Quarter dilatation velocity (pix/ms)  Half dilatation time (ms)  Line integral | 239.7952083  250.1666667  262.7806250  0.0902316  0.0549163  479.5217391  3521.39 | 27.0105948  30.6714458  31.0681464  0.0128567  0.0036267  81.5422833  1.7194325 |
| **37** | sleep deprivation | Initial diameter (pix)  Latency (ms)  Acceleration (ms)  Contraction velocity (pix/ms)  Quarter dilatation velocity (pix/ms)  Half dilatation time (ms)  Line integral | 231.7497959  257.4565217  263.2608511  0.0870534  0.0531394  482.2380952  3520.03 | 24.8105078  32.4226989  34.8173068  0.0130549  0.0060704  75.7865454  2.9994574 |
| **37** | alcohol exposure | Initial diameter (pix)  Latency (ms)  Acceleration (ms)  Contraction velocity (pix/ms)  Quarter dilatation velocity (pix/ms)  Half dilatation time (ms)  Line integral | 238.1137500  245.8593750  270.4670313  0.0916053  0.0534357  526.9375000  3521.52 | 25.1397170  24.3505270  34.0321837  0.0144724  0.0041740  109.5363787  1.4609534 |
| **38** | baseline | Initial diameter (pix)  Latency (ms)  Acceleration (ms)  Contraction velocity (pix/ms)  Quarter dilatation velocity (pix/ms)  Half dilatation time (ms)  Line integral | 199.5287500  219.4126984  230.9188889  0.0656156  0.0488413  386.2903226  3515.59 | 18.1758780  17.5991522  16.9328126  0.0173893  0.0078033  142.0226704  2.0262451 |
| **38** | sleep deprivation | Initial diameter (pix)  Latency (ms)  Acceleration (ms)  Contraction velocity (pix/ms)  Quarter dilatation velocity (pix/ms)  Half dilatation time (ms)  Line integral | 225.5760938  222.7500000  241.5026984  0.0736048  0.0519623  422.0322581  3515.90 | 23.6782212  16.2079737  20.0804446  0.0173190  0.0059013  126.2507222  1.9120439 |
| **38** | alcohol exposure | Initial diameter (pix)  Latency (ms)  Acceleration (ms)  Contraction velocity (pix/ms)  Quarter dilatation velocity (pix/ms)  Half dilatation time (ms)  Line integral | 232.2326563  216.0781250  238.8800000  0.0790239  0.0557542  413.3000000  3516.70 | 20.4209060  13.1695954  18.8957235  0.0186510  0.0067855  113.5492576  1.7022725 |
| **39** | baseline | Initial diameter (pix)  Latency (ms)  Acceleration (ms)  Contraction velocity (pix/ms)  Quarter dilatation velocity (pix/ms)  Half dilatation time (ms)  Line integral | 246.2923437  217.2500000  247.1621875  0.1201017  0.0749310  502.7096774  3530.63 | 38.2719737  14.8772757  22.0404319  0.0160299  0.0055856  116.2377985  2.3092843 |
| **39** | sleep deprivation | Initial diameter (pix)  Latency (ms)  Acceleration (ms)  Contraction velocity (pix/ms)  Quarter dilatation velocity (pix/ms)  Half dilatation time (ms)  Line integral | 227.3946875  215.1250000  246.0384375  0.1197969  0.0721702  508.1875000  3530.64 | 36.8421838  14.0627844  25.6070177  0.0156857  0.0057526  101.4840475  2.6104384 |
| **39** | alcohol exposure | Initial diameter (pix)  Latency (ms)  Acceleration (ms)  Contraction velocity (pix/ms)  Quarter dilatation velocity (pix/ms)  Half dilatation time (ms)  Line integral | 241.7710938  216.2500000  248.3176563  0.1171579  0.0739163  482.4375000  3530.06 | 36.4095013  13.5950505  20.2256192  0.0174247  0.0052862  101.5898812  2.8944108 |
| **40** | baseline | Initial diameter (pix)  Latency (ms)  Acceleration (ms)  Contraction velocity (pix/ms)  Quarter dilatation velocity (pix/ms)  Half dilatation time (ms)  Line integral | 238.7138636  237.8863636  252.8495455  0.0859684  0.0659704  336.6363636  3522.50 | 33.5751574  21.2035713  17.1644585  0.0074052  0.0049713  31.9076363  2.4896199 |
| **40** | sleep deprivation | Initial diameter (pix)  Latency (ms)  Acceleration (ms)  Contraction velocity (pix/ms)  Quarter dilatation velocity (pix/ms)  Half dilatation time (ms)  Line integral | 236.3985714  238.5178571  260.5857143  0.0839666  0.0615634  345.0000000  3522.10 | 23.7006639  16.6405759  24.0156404  0.0138718  0.0084252  34.4831975  3.8999546 |
| **40** | alcohol exposure | Initial diameter (pix)  Latency (ms)  Acceleration (ms)  Contraction velocity (pix/ms)  Quarter dilatation velocity (pix/ms)  Half dilatation time (ms)  Line integral | 252.1143750  229.6250000  257.0193750  0.1026384  0.0733694  371.6875000  3526.16 | 32.1993686  15.9398672  22.3932856  0.0166910  0.0084009  53.2609256  3.9359170 |
| **41** | baseline | Initial diameter (pix)  Latency (ms)  Acceleration (ms)  Contraction velocity (pix/ms)  Quarter dilatation velocity (pix/ms)  Half dilatation time (ms)  Line integral | 249.0110938  224.3906250  251.6403125  0.0887320  0.0678424  352.6250000  3522.58 | 24.4777764  15.7798517  27.5222630  0.0132277  0.0066792  39.9251283  2.2500482 |
| **41** | sleep deprivation | Initial diameter (pix)  Latency (ms)  Acceleration (ms)  Contraction velocity (pix/ms)  Quarter dilatation velocity (pix/ms)  Half dilatation time (ms)  Line integral | 246.3537500  223.5000000  251.1657813  0.0893820  0.0667643  359.0937500  3522.25 | 27.4082577  15.5777619  26.8311539  0.0177419  0.0089917  61.6608025  3.0909403 |
| **41** | alcohol exposure | Initial diameter (pix)  Latency (ms)  Acceleration (ms)  Contraction velocity (pix/ms)  Quarter dilatation velocity (pix/ms)  Half dilatation time (ms)  Line integral | 249.2810938  216.7812500  245.0675000  0.0962714  0.0706134  371.5625000  3525.68 | 26.6151266  16.3585507  27.3433375  0.0207258  0.0097704  70.5816818  2.0346654 |
| **42** | baseline | Initial diameter (pix)  Latency (ms)  Acceleration (ms)  Contraction velocity (pix/ms)  Quarter dilatation velocity (pix/ms)  Half dilatation time (ms)  Line integral | 218.1754688  240.6129032  266.9868852  0.0818109  0.0598191  371.1000000  3519.97 | 20.7549304  26.8635623  21.6260189  0.0138753  0.0066263  101.7728777  1.7286309 |
| **42** | sleep deprivation | Initial diameter (pix)  Latency (ms)  Acceleration (ms)  Contraction velocity (pix/ms)  Quarter dilatation velocity (pix/ms)  Half dilatation time (ms)  Line integral | 200.4800000  250.3000000  270.3380000  0.0718461  0.0548615  314.7000000  3519.68 | 15.0596327  22.9280339  22.4453788  0.0044862  0.0035590  35.2257382  4.1221955 |
| **42** | alcohol exposure | Initial diameter (pix)  Latency (ms)  Acceleration (ms)  Contraction velocity (pix/ms)  Quarter dilatation velocity (pix/ms)  Half dilatation time (ms)  Line integral | 228.2896429  239.9464286  266.9408929  0.0905746  0.0626746  403.7142857  3522.33 | 23.8065824  19.0362522  19.5851935  0.0185569  0.0079649  96.1944459  3.2972555 |
| **43** | baseline | Initial diameter (pix)  Latency (ms)  Acceleration (ms)  Contraction velocity (pix/ms)  Quarter dilatation velocity (pix/ms)  Half dilatation time (ms)  Line integral | 323.2078125  217.2031250  259.2548438  0.1029454  0.0700920  459.0000000  3524.68 | 29.0972712  12.1200080  26.6409652  0.0112460  0.0051809  56.9483197  3.3602559 |
| **43** | sleep deprivation | Initial diameter (pix)  Latency (ms)  Acceleration (ms)  Contraction velocity (pix/ms)  Quarter dilatation velocity (pix/ms)  Half dilatation time (ms)  Line integral | 318.6971875  227.4375000  267.7929688  0.0991665  0.0651554  492.4666667  3523.51 | 32.1510830  17.0906779  27.9096143  0.0098658  0.0065308  71.3362690  2.1900737 |
| **43** | alcohol exposure | Initial diameter (pix)  Latency (ms)  Acceleration (ms)  Contraction velocity (pix/ms)  Quarter dilatation velocity (pix/ms)  Half dilatation time (ms)  Line integral | 338.2839062  228.9843750  269.2485938  0.0965832  0.0600385  525.6875000  3522.36 | 31.3604598  15.2996443  31.7320782  0.0184730  0.0084810  112.6935548  3.6068045 |
| **44** | baseline | Initial diameter (pix)  Latency (ms)  Acceleration (ms)  Contraction velocity (pix/ms)  Quarter dilatation velocity (pix/ms)  Half dilatation time (ms)  Line integral | 187.9668750  226.7031250  240.4268750  0.0843492  0.0551119  393.7500000  3521.74 | 21.9169143  19.6522786  28.3542770  0.0109226  0.0050683  37.9146912  2.5283795 |
| **44** | sleep deprivation | Initial diameter (pix)  Latency (ms)  Acceleration (ms)  Contraction velocity (pix/ms)  Quarter dilatation velocity (pix/ms)  Half dilatation time (ms)  Line integral | 193.1140625  218.5468750  241.2185938  0.0977893  0.0585227  441.5161290  3525.55 | 34.0407331  18.4837378  31.2356210  0.0234805  0.0090718  96.7826265  5.6199729 |
| **44** | alcohol exposure | Initial diameter (pix)  Latency (ms)  Acceleration (ms)  Contraction velocity (pix/ms)  Quarter dilatation velocity (pix/ms)  Half dilatation time (ms)  Line integral | 195.6209375  212.3437500  233.5967188  0.1002706  0.0612152  453.0625000  3525.47 | 27.8036653  15.7552901  25.9278504  0.0155131  0.0049730  80.6363333  1.6484374 |
| **45** | baseline | Initial diameter (pix)  Latency (ms)  Acceleration (ms)  Contraction velocity (pix/ms)  Quarter dilatation velocity (pix/ms)  Half dilatation time (ms)  Line integral | 202.4019355  206.9677419  213.5433871  0.0798142  0.0635031  331.5172414  3520.38 | 20.7329593  14.8809128  16.1204752  0.0122989  0.0061217  40.6453387  1.6623219 |
| **45** | sleep deprivation | Initial diameter (pix)  Latency (ms)  Acceleration (ms)  Contraction velocity (pix/ms)  Quarter dilatation velocity (pix/ms)  Half dilatation time (ms)  Line integral | 200.3034426  209.0983607  215.3791667  0.0813229  0.0608563  358.7586207  3520.34 | 24.8060475  14.4230197  17.0957975  0.0131029  0.0049263  68.1863572  2.7096831 |
| **45** | alcohol exposure | Initial diameter (pix)  Latency (ms)  Acceleration (ms)  Contraction velocity (pix/ms)  Quarter dilatation velocity (pix/ms)  Half dilatation time (ms)  Line integral | 184.1534426  210.9180328  218.8893333  0.0691839  0.0544064  322.2222222  3516.86 | 20.0352567  12.8922394  16.7446332  0.0106776  0.0051707  47.5492462  1.3602810 |
| **46** | baseline | Initial diameter (pix)  Latency (ms)  Acceleration (ms)  Contraction velocity (pix/ms)  Quarter dilatation velocity (pix/ms)  Half dilatation time (ms)  Line integral | 246.9395313  213.0312500  237.1843750  0.0979713  0.0721847  356.5312500  3526.51 | 30.5341832  15.8254053  23.2588068  0.0099714  0.0047174  40.2894168  2.1070487 |
| **46** | sleep deprivation | Initial diameter (pix)  Latency (ms)  Acceleration (ms)  Contraction velocity (pix/ms)  Quarter dilatation velocity (pix/ms)  Half dilatation time (ms)  Line integral | 240.8985938  219.1111111  237.1029688  0.0946453  0.0724239  337.0000000  3524.41 | 25.7581480  17.4412480  18.5649853  0.0121821  0.0059012  37.1175738  2.0769229 |
| **46** | alcohol exposure | Initial diameter (pix)  Latency (ms)  Acceleration (ms)  Contraction velocity (pix/ms)  Quarter dilatation velocity (pix/ms)  Half dilatation time (ms)  Line integral | 253.8131250  215.6031746  237.1850794  0.1011430  0.0755916  355.5483871  3527.23 | 27.9126123  10.5685774  18.8036696  0.0123140  0.0060297  39.5332097  2.3290157 |
| **47** | baseline | Initial diameter (pix)  Latency (ms)  Acceleration (ms)  Contraction velocity (pix/ms)  Quarter dilatation velocity (pix/ms)  Half dilatation time (ms)  Line integral | 238.5287500  265.3492063  299.2295313  0.0838303  0.0538071  492.2812500  3519.96 | 25.7558306  26.7130261  32.2630591  0.0164702  0.0049407  107.0366305  2.7420032 |
| **47** | sleep deprivation | Initial diameter (pix)  Latency (ms)  Acceleration (ms)  Contraction velocity (pix/ms)  Quarter dilatation velocity (pix/ms)  Half dilatation time (ms)  Line integral | 231.5859375  253.6250000  295.7525000  0.0808447  0.0510602  484.8333333  3518.49 | 26.5499161  26.4620123  31.1940468  0.0189205  0.0052236  108.5138696  2.9375413 |
| **47** | alcohol exposure | Initial diameter (pix)  Latency (ms)  Acceleration (ms)  Contraction velocity (pix/ms)  Quarter dilatation velocity (pix/ms)  Half dilatation time (ms)  Line integral | 228.9520313  255.0476190  294.0032813  0.0749248  0.0497090  461.8387097  3517.01 | 17.9545391  21.1678610  29.6620206  0.0176060  0.0061187  97.5510280  2.1555418 |
| **48** | baseline | Initial diameter (pix)  Latency (ms)  Acceleration (ms)  Contraction velocity (pix/ms)  Quarter dilatation velocity (pix/ms)  Half dilatation time (ms)  Line integral | 268.3114063  201.7500000  225.0557813  0.1380508  0.0843746  483.4375000  3538.36 | 39.5798625  14.8580586  27.0965980  0.0147076  0.0043948  79.4552634  1.8067018 |
| **48** | sleep deprivation | Initial diameter (pix)  Latency (ms)  Acceleration (ms)  Contraction velocity (pix/ms)  Quarter dilatation velocity (pix/ms)  Half dilatation time (ms)  Line integral | 272.2365625  202.8593750  221.4321875  0.1307096  0.0800013  496.3437500  3535.30 | 40.4040600  13.6391909  21.2358934  0.0174381  0.0043898  105.8980070  2.0092351 |
| **48** | alcohol exposure | Initial diameter (pix)  Latency (ms)  Acceleration (ms)  Contraction velocity (pix/ms)  Quarter dilatation velocity (pix/ms)  Half dilatation time (ms)  Line integral | 273.7357813  201.1562500  223.0059375  0.1278508  0.0782944  476.1250000  3533.87 | 38.4930622  13.5836255  25.4028642  0.0150482  0.0052257  95.4197910  3.0051438 |
| **49** | baseline | Initial diameter (pix)  Latency (ms)  Acceleration (ms)  Contraction velocity (pix/ms)  Quarter dilatation velocity (pix/ms)  Half dilatation time (ms)  Line integral | 212.3343750  247.3281250  290.2701562  0.0863696  0.0586580  404.1250000  3520.34 | 21.6360214  27.5836705  22.6885135  0.0141518  0.0054269  49.4944281  2.6686087 |
| **49** | sleep deprivation | Initial diameter (pix)  Latency (ms)  Acceleration (ms)  Contraction velocity (pix/ms)  Quarter dilatation velocity (pix/ms)  Half dilatation time (ms)  Line integral | 252.7868966  252.6610169  303.0045763  0.0999423  0.0607947  532.8928571  3523.52 | 34.1569322  26.3946247  29.6805251  0.0233041  0.0063722  123.6335757  2.3309069 |
| **49** | alcohol exposure | Initial diameter (pix)  Latency (ms)  Acceleration (ms)  Contraction velocity (pix/ms)  Quarter dilatation velocity (pix/ms)  Half dilatation time (ms)  Line integral | 234.0010345  260.1551724  304.1000000  0.0913317  0.0595399  466.2592593  3522.74 | 24.5976582  23.0588886  31.9617076  0.0184395  0.0046752  95.0724024  2.3460190 |
| **50** | baseline | Initial diameter (pix)  Latency (ms)  Acceleration (ms)  Contraction velocity (pix/ms)  Quarter dilatation velocity (pix/ms)  Half dilatation time (ms)  Line integral | 234.9581250  232.6406250  264.6926563  0.0764871  0.0447322  513.4375000  3517.66 | 21.5322123  16.1297981  32.1611551  0.0149223  0.0040666  128.6553011  1.1372756 |
| **50** | sleep deprivation | Initial diameter (pix)  Latency (ms)  Acceleration (ms)  Contraction velocity (pix/ms)  Quarter dilatation velocity (pix/ms)  Half dilatation time (ms)  Line integral | 225.2046875  243.5079365  272.6832813  0.0688296  0.0399119  497.4827586  3516.28 | 22.3284111  22.7240621  34.5780288  0.0200415  0.0046764  156.1853646  1.0549989 |
| **50** | alcohol exposure | Initial diameter (pix)  Latency (ms)  Acceleration (ms)  Contraction velocity (pix/ms)  Quarter dilatation velocity (pix/ms)  Half dilatation time (ms)  Line integral | 257.4112500  244.4843750  273.2993651  0.0759545  0.0413237  550.5925926  3518.13 | 26.5070679  16.7312958  30.8604539  0.0206280  0.0042348  176.3016838  1.7719050 |
